# Cancer mortality and morbidity among patients with schizophrenia: A hospital-based cohort study, 1992-2020

**DOI:** 10.1192/j.eurpsy.2025.1468

**Published:** 2025-08-26

**Authors:** M. Drevinskaite, A. Kaceniene, R. Stukas, A. Germanavicius, G. Smailyte

**Affiliations:** 1 Department of Public Health, Institute of Health Sciences, Vilnius University, Faculty of Medicine; 2 Laboratory of Cancer Epidemiology, National Cancer Institute; 3 Clinic of Psychiatry, Institute of Clinical Medicine, Vilnius University, Faculty of Medicine; 4 Department of Psychiatry, Republican Vilnius Psychiatric Hospital, Vilnius, Lithuania

## Abstract

**Introduction:**

The inconsistency in cancer incidence and mortality rates in terms of cancer site reported among patients with schizophrenia has been an interesting topic in epidemiology, and additional studies are necessary to gain a more comprehensive understanding.

**Objectives:**

Due to the inconsistency of the evidence about the cancer risk among patients with schizophrenia, the aim of this study was to analyse cancer mortality and morbidity in patients with schizophrenia treated in a single centre in Lithuania during the study period of 1992-2020.

**Methods:**

A retrospective cohort study was conducted in Vilnius Republican Psychiatric Hospital, the biggest specialised psychiatric hospital in Lithuania, with approximately 5000 hospital admissions annually. The patients’ cohort was established by identifying all patients with the diagnosis of schizophrenia (ICD-10 code F20) in the hospital database from 1 January 1992 until 31 December 2017. The cancer cases and cancer deaths in the cohort were identified in the Lithuanian Cancer Register through linkage procedures. The analysis of risk was based on a comparison of observed and expected numbers of cancers and deaths. Expected number of cancer cases were calculated by multiplication of the exact person-years under observation in the cohort by sex, calendar year and a 5-year age-group-specific national incidence and mortality rate. All statistical analyses were carried out using STATA 15 statistical software.

**Results:**

During the follow-up, out of 8553 patients, 673 cases of cancer were diagnosed in both sexes. Statistically significantly lower risk for overall cancer incidence was observed in men (SIR 0.74, 95% CI 0.66-0.83), but not in women (SIR 1.07, 95% CI 0.97-1.18). We observed lower risk for pancreatic cancer (SIR 0.36, 95% CI 0.14-0.96), non-melanoma skin cancer (SIR 0.54, 95% CI 0.33-0.88) and prostate cancer (SIR 0.69, 95% CI 0.55-0.87) in men and higher risk for malignant neoplasm of liver (SIR 2.58, 95% CI 1.53-4.36) and skin melanoma (SIR 2.03, 95% CI 1.12-3.66) in men and for breast cancer (SIR 1.38, 95% CI 1.14-1.66) and corpus uteri cancer (SIR 1.56, 95% CI 1.18-2.07) in women (Table 1). Statistically significant lower overall cancer mortality risk was observed in men (SMR 0.82, 95% CI 0.70-0.96), while in the women’s group, risk of cancer deaths was significantly higher compared to the general population (SMR 1.28, 95% CI 1.11-1.48).

**Conclusions:**

The current results of our study indicate lower risk of overall cancer incidence and mortality in male patients with schizophrenia, while female patients had a higher mortality risk, alongside variations in the risk of different cancer types. This information is important not only for patients, but for healthcare specialists to develop effective disease-specific preventive interventions and programmes.

**Disclosure of Interest:**

None Declared

